# Heart failure and trimethylamine N‐oxide: time to transform a ‘gut feeling’ in a fact?

**DOI:** 10.1002/ehf2.14205

**Published:** 2022-10-13

**Authors:** Giulia Crisci, Muhammad Zubair Israr, Antonio Cittadini, Eduardo Bossone, Toru Suzuki, Andrea Salzano

**Affiliations:** ^1^ Department of Translational Medical Sciences Federico II University Via S Pansini 5 Naples 80131 Italy; ^2^ Italian Clinical Outcome Research and Reporting Program (I‐CORRP) Naples 80131 Italy; ^3^ Department of Cardiovascular Sciences University of Leicester and NIHR Leicester Biomedical Research Centre Groby road Leicester LE3 9QP United Kingdom; ^4^ Department of Public Health Federico II University Naples 80138 Italy; ^5^ IRCCS Synlab SDN Diagnostic and Nuclear Research Institute Via E Gianturco 113 Naples 80143 Italy

In the last few years, in the context of a growing interest in investigating novel pathways involved in heart failure (HF) pathophysiology, the association between the gastrointestinal (GI) system and HF represents an important model of attention, the so called ‘gut hypothesis’.^1^, ^2^, ^3^ Despite being classically identified as a ‘simple’ intestinal dysfunction, the main hypothesis is currently focused on the role of inflammation and oxidative stress as a consequence of the intestinal wall ischaemia and/or congestion induced by HF, determining a gut barrier dysfunction and resulting in an increased gut bacterial translocation.^1^, ^2^, ^3^ With this in mind, two main mechanisms have been proposed to link gut dysfunction and HF; (i) metabolism dependent, via gut‐derived metabolites entering the systemic circulation and exerting pro‐atherogenic effects and pro‐inflammatory effects and (ii) metabolism independent, via bacterial components (e.g. lipopolysaccharides and endotoxins) translocating in the systemic circulation and contributing to the systemic inflammatory state with its well‐known negative effects on HF.^4^

To date, most of the research has identified a choline and L‐carnitine metabolic by‐product, trimethylamine N‐oxide (TMAO), derived by the gut microbiota from the precursor trimethylamine (TMA) and subsequent oxidation via the liver enzyme flavin‐containing monooxygenase 3 (FMO3) (see *Figure* *1*), as the key useful prognostic biomarker in several cardiovascular diseases (e.g. coronary artery disease, acute myocardial infarction, and HF), with an interesting role in risk stratification.^5^, ^6^ Notably, TMAO, produced through the anaerobic metabolism of choline and carnitine containing molecules, is widely considered as the possible missing link between the consumption of a Western diet and the well‐known increased cardiovascular diseases risk observed in the Western population.^7^ Indeed, TMAO is produced through the anaerobic metabolism of choline and L‐carnitine, of which eggs and red meat are rich in the Western diet (i.e., based on high fat foods), and diet can be considered as one the of most important factors affecting the gut microbiota composition.^8^

**Figure 1:**
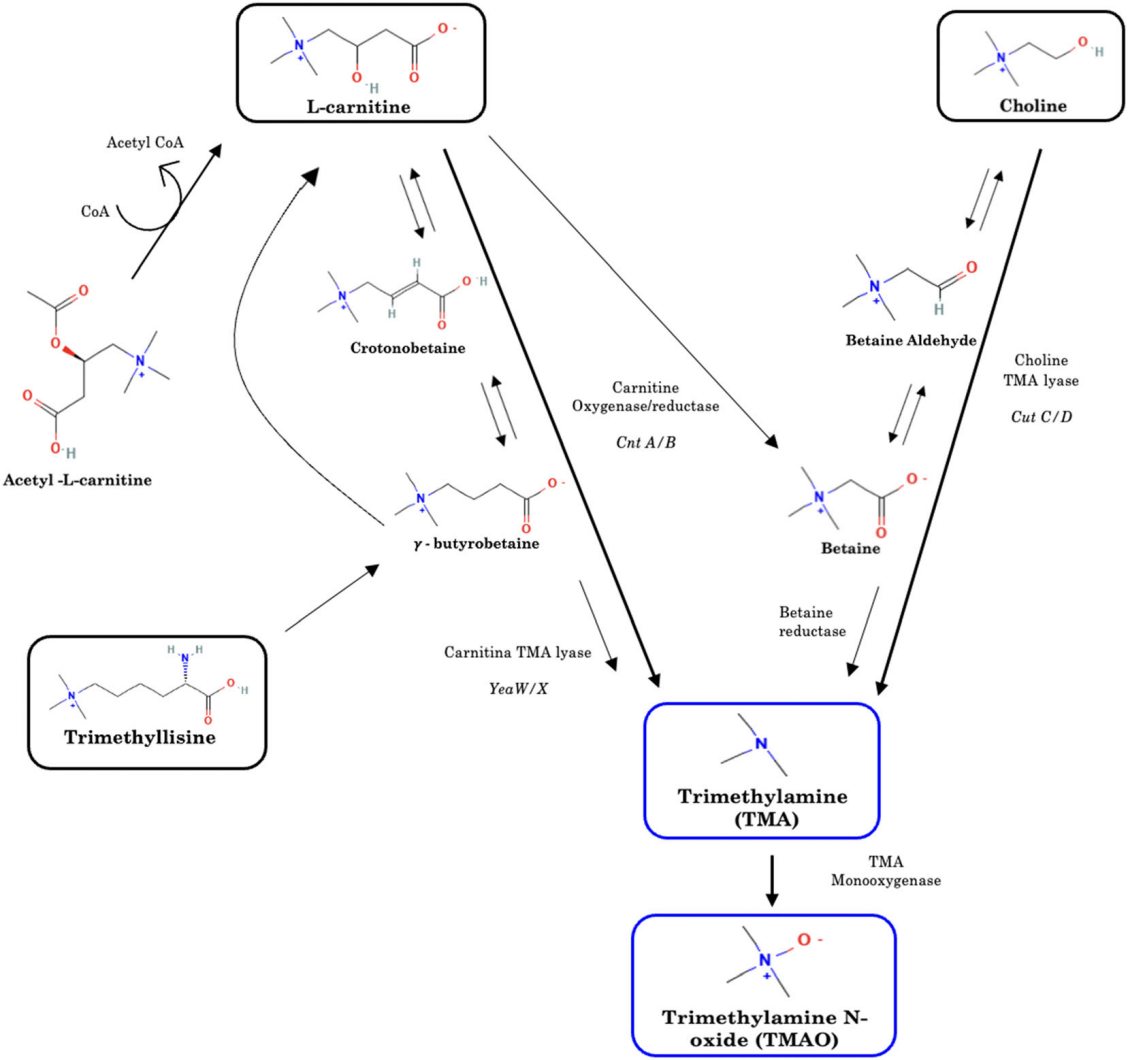
Multiple pathways and metabolites involved in the synthesis of trimethylamine N‐oxide (TMAO).

Amongst the cardiovascular diseases, HF represents a widely investigated disease for association with the gut, with evidence available in both reduced (HFrEF)^9^, ^10^, ^11^, ^12^, ^13^, ^14^, ^15^, ^16^, ^17^, ^18^, ^19^, ^20^, ^21^ and preserved (HFpEF)^22^, ^23^, ^24^, ^25^, ^26^, ^27^ phenotypes, as well as in acute^12^, ^13^, ^28^, ^29^, ^30^ and chronic setting^9^, ^10^, ^11^, ^14^, ^15^, ^16^, ^17^, ^18^, ^19^, ^20^, ^21^, ^22^, ^23^, ^24^, ^25^, ^27^ (see *Table* *1*). From a pathophysiological point of view, TMAO pathway affects HF in different ways.^1^, ^3^ First, TMAO increases the risk of conditions determining HF (i.e. cardiac ischemic diseases), through its pro‐atherosclerotic effects mediated by an increase in the expression of macrophage scavenger receptors with development of foam cells in the arterial wall, increasing thrombosis, increasing platelet reactivity, and causing endothelial dysfunction.^1^, ^3^ Second, TMAO increases HF susceptibility, directly acting on myocardial remodelling and fibrosis; in addition, it has been speculated that TMAO, being an organic osmolyte, altered cellular osmosis; lastly, it has been showed that TMAO worsens cardiomyocyte contractility, by acting on calcium cellular fluxes.^1^, ^3^ Worthy to be mentioned is the relationship between TMAO and renal function, with a possible effect on renal fibrosis and tubular injury further aggravating HF clinic.^1^, ^3^

**Table 1:** Main reports for associations between trimethylamine N‐oxide and outcome in heart failure patients

First author; year of publication	Location	Study population	Follow‐up length	Main findings	Trimethylamine N‐oxide levels (μmol/L)
Tang WH 2014^9^	USA	CHF, *N* = 720	5 years	TMAO levels are associated with all‐cause mortality	5.0 (3.0–8.5)
Tang WH 2015^10^	USA	CHF, *N* = 112	5 years	TMAO levels are associated with all‐cause mortality and heart transplantation	5.8 (3.6–12.1)
Trøseid M 2015^11^	Norway	CHF *N* = 115	5.2 years	TMAO levels are associated with all‐cause mortality and heart transplantation	13.5 ± 18.5 (CAD), 7.1 ± 5.6 (DCM)
Suzuki T 2016^12^	UK	AHF, *N* = 972	1 years	TMAO levels are associated with all‐cause mortality and a composite mortality/rehospitalization	5.6 (3.4–10.5)
Schuett K 2017^22^	Germany	CHF (pEF and rEF), *N* = 823	9.7 years	TMAO levels are associated with all‐cause mortality and cardiovascular mortality	4.7 (3.4–6.8) rEF, 4.7 (3.2–6.9) pEF
Hayashi T 2018^13^	Japan	Decomp HF, *N* = 22	Cross‐sectional	TMAO levels (during decompensation and during compensation phases) and gut microbiome composition were altered compared with control subjects	17.3 ± 11.7 (Decomp), 17.7 ± 12.6 (Comp)
Salzano A 2019^23^	UK	CHF (pEF and rEF), pEF = 118 *vs* rEF = 38 *vs* C = 40, *N* = 196	5 years	TMAO levels are associated with mortality in pEF Use of levels of TMAO for risk stratification of long‐term mortality in pEF	6.6 (4.3–12.2) pEF, 8.4 (3.7–13.8) rEF
Suzuki T 2019^14^	11 European countries	Worsening or new‐onset HF, *N* = 2234	3 years	TMAO levels are associated with all‐cause mortality and a composite of mortality/rehospitalization	5.9 (3.6–10.8)
Yazaki Y 2019^31^	11 European countries	Worsening or new‐onset HF, *N* = 2234	2 years	TMAO levels of HF patients differed by region TMAO levels associated with risk of mortality CE > NW/S	6.2 (4.8–7.8) CE, 7.2 (5.5–8.8) NW, 6.5 (5.0–8.2) S
Zhou X 2020^16^	China	HFrEF after MI, *N* = 1208	4 years	TMAO levels are associated with major adverse cardiac events (MACE): all‐cause mortality, HF rehospitalization, or recurrent MI, and all‐cause mortality	4.5
Yazaki Y 2020^28^	UK	AHF, *N* = 1087	1 year	TMAO levels are associated with a composite of all‐cause mortality and/or rehospitalization	5.2–22.8 (Japanese), 3.6–10.8 (Caucasian), 3.1–8.4 (South Asian)
Guo F 2020^24^	China	HFpEF, *N* = 228	5 years	TMAO levels are independent predictor of new onset HF TMAO levels are independent risk factor of renal dysfunction	12.65 (9.32–18.66)
Emoto T 2021^25^		CHF (pEF and rEF), CHF = 22 *vs* C = 11, *N* = 33		TMAO levels are increased in Japanese HF compared to Caucasian/South Asian HF abundance of cntA/B positively correlated with TMAO	4.5–34.5
Papandreou C 2021^17^	Spain	CHF (pEF and rEF), AF = 509 *vs* C = 618, CHF = 326 *vs* C = 426, *N* = 1879	10 years	TMAO levels are not associated with AF and HF incidence	3.0–8.5
Kinugasa Y 2021^26^	Japan	HFpEF, *N* = 146	5 years	TMAO levels are associated with a composite endpoint of cardiac mortality and hospitalization for HF	20.37
Israr MZ 2021^30^	UK	AHF, *N* = 806	1 years	TMAO levels are associated with all‐cause mortality and a composite of all‐cause mortality and/or rehospitalization caused by HF	10.2 (5.8–18.7)
Dong Z 2021^27^	China	HFpEF, CHF = 61 *vs* C = 57, *N* = 118	1 years	TMAO levels are independent risk factor for HFpEF	6.84
Yuzefpolskaya M 2021^18^	USA	CHF, *N* = 341	2 years + 8 months	TMAO levels increased with HF severity and were similarly elevated, long term after LVAD and HT. TMAO levels positively related to biomarkers of inflammation (TNF‐α and ET‐1), endotoxemia (sCD14), and oxidative stress	6.96 (HF), 5.81 (LVAD), 5.35 (HT)
Mollar A 2021^19^	Spain	Decomp HF, *N* = 102	1 years	TMAO related with recent HF	
Wargny M 2022^32^	France	AHF, AHF = 209 *vs* C = 1140, *N* = 1468	7.3 years	TMAO is not associated with occurrence of HF requiring hospitalization (HFrH) and composite event HFrH and/or cardiovascular mortality and all‐cause mortality.	8.8 (5.3–17.0)
Li N 2022^29^	China	AMI and HF, *N* = 985	1 years	TMAO levels independently correlated with poor prognosis (i.e.: MACE, and all‐cause mortality) and recurrence of MI in patients with AMI complicated by HF, especially in those with higher hsCRP levels TMAO the difference for rehospitalization due to HF is not statistically significant	6.7 (4.0, 11.7)
Wei H 2022^20^	China	HFrEF, *N* = 955	8 years	TMAO levels are associated with the composite outcome of cardiovascular death or heart transplantation	2.52 (1.18–4.06)
Israr MZ 2022^21^	UK	HFrEF, *N* = 1783	3 years	TMAO levels are associated with the composite outcome of HF hospitalization or death at 3 years	6.4 (3.9–11.6)

Abbreviations: AF, atrial fibrillation; AMI, acute myocardial infarction; AHF, acute heart failure; BUN, blood urea nitrogen; C, controls; CAD, coronary artery disease; CE, Central/Eastern group (Germany, Poland, Serbia, and Slovenia); CHF, chronic heart failure; cntA/B, carnitine oxygenase/reductase; Comp, compensated; DCM, dilated cardiomyopathy; Decomp, decompensated; ET‐1, endothelin‐1; HF, heart failure; HFpEF, heart failure with preserved ejection fraction; HFrEF, heart failure with reduced ejection fraction; hsCRP, high‐sensitivity C‐reactive protein; HT, heart transplant; LVAD, left ventricular assist device; MACE, major adverse cardiac events; MI, myocardial infarction; NT‐proBNP, N‐terminal pro‐brain type natriuretic peptide; NYHA, New York Heart Association; NW, the Northern/Western group (France, Netherlands, Norway, Sweden, and United Kingdom); S, Southern group (Greece and Italy); sCD14, soluble CD14; TMAO, trimethylamine N‐oxide; TNF‐α, tumour necrosis factor alpha. TMAO levels expressed as mean ± standard deviation or median (interquartile range).

From a clinical point of view, since the first investigations regarding the association between TMAO and HFrEF as of about 10 years ago,^9^ several studies have demonstrated that TMAO levels were higher in CHF when compared with healthy controls showing associations between TMAO and clinical and biochemical parameters (i.e., renal function, age, comorbidities, and CRP), severity of disease (NYHA classes),^15^, ^18^ and clinical outcomes (death, HF hospitalization, composite).^9^, ^10^, ^11^, ^12^, ^13^, ^14^, ^15^, ^16^, ^17^, ^18^, ^19^, ^20^, ^21^ As a prototype, in the BIOSTAT‐CHF (BIOlogy Study to TAilored Treatment in Chronic Heart Failure) population, in which 2234 patients with new‐onset or progressively worsening HF have been investigated, TMAO levels were associated with adverse outcomes (mortality and/or rehospitalisation) at different time‐points (1, 2, and 3 years).^14^ Despite being the most prevalent phenotype in HF, only a few studies (when compared with the numerosity of study about HFrEF phenotype) have investigated the association of TMAO with outcome in HFpEF subjects.^22^, ^23^, ^24^, ^25^, ^26^, ^27^ The Developing Imaging And plasMa biOmarkers iN Describing Heart Failure With Preserved Ejection Fraction (DIAMONDHFpEF) cohort showed that TMAO was associated with adverse outcome in HFpEF and that its use allows a better stratification of HFpEF patients when used in combination with BNP.^23^ Considering that natriuretic peptides are not as highly elevated in HFpEF compared with HFrEF, in this cohort elevated TMAO levels improved risk stratification of patients in which BNP levels show equivocal levels.

The role of TMAO in risk stratification is not limited to the chronic setting; indeed, TMAO showed its value also in the acute setting, in which elevated levels (except in one investigation)^32^ showed to be associated with a poor prognosis.^12^, ^13^, ^28^, ^29^, ^30^ For instance, in a cohort of 972 acute HF patients, TMAO levels were associated with a more reduced left ventricular ejection fraction, a more advanced clinical status (i.e. NYHA classes III‐IV), and with poor outcome.^12^ Interestingly, even in this setting, TMAO showed to improve risk stratification when combined with clinical scores [i.e., Acute Decompensated Heart Failure National Registry (ADHERE), Organized Program to Initiate Lifesaving Treatment in Hospitalized Patients with Heart Failure (OPTIMIZE‐HF), Get With The Guidelines‐Heart Failure (GWTG‐HF)], and with NT‐proBNP.^12^

Finally, when all these findings have been analysed in three independent meta‐analyses, consistent results show elevated TMAO levels are associated with poor prognosis (i.e., MACE and all‐cause mortality) in HF patients, even when adjusted for multiple confounders.^33^, ^34^, ^35^

In this context, in the present issue of the *ESC Heart Failure* journal, Li et al. showed that elevated TMAO levels were associated with poor prognosis (i.e. all‐cause death and recurrence of myocardial infarction) in patients with acute myocardial infarction complicated by HF,^29^ further validating current understanding.^12^, ^36^ Specifically, the authors found that patients with higher sensitivity C‐reactive protein (hsCRP) levels resulted in a further significant stratification of TMAO by tertiles for MACE. This is of great interest, because it has been suggested that, mechanistically, TMAO induces HF and myocardial ischaemia via mechanisms including inflammation, mitochondrial dysfunction, production of oxygen‐free radicals, and myocardial fibrosis.^1^ These mechanisms play an important role in the development and progression of plaques, lipid deposition to plaque rupture, and its eventual complications.^37^ In addition, it has been shown that bowel thickness was directly correlated with circulating hsCRP levels,^1^ further supporting the role of a bacterial translocation in HF and their association with systemic inflammation. When investigating the role of TMAO considering its involvement with CRP, current evidence suggests that TMAO activates different signalling pathways; for example, the nuclear factor‐ᴋB signalling pathway to induce inflammation by TMAO^38^ or by activating the nucleotide‐binding oligomerization domain‐like receptor family pyrin domain‐containing 3 (NLRP3) inflammasome and subsequently triggering systemic inflammation.^39^ Intriguingly, the present paper showed that increasing hsCRP might increase the association of TMAO levels with HF outcome, suggesting a potential synergistic effect between TMAO and inflammation.

Another finding of interest of the present investigation, considering the positive dose‐dependent association between TMAO plasma levels and increased cardiovascular risk and mortality,^33^ is the evidence that TMAO levels were significantly more increased in HFrEF than in HFpEF, reflecting the different pathophysiology underpinning the two phenotypes.

One important limitation for the present study is the lack of dietary information. Indeed, adjusting TMAO data for dietary information is paramount to ascribing the ‘true’ normal ranges, association with HF/MI and intervention in a ‘real‐world’ scenario. To elaborate, TMAO levels in Caucasian patients showed increased association with adverse outcomes, but not in non‐Caucasian patients despite any differences between ethnicity and association with outcome, suggesting that dietary sources could be the contributing factor.^28^ Furthermore, when a diet‐based categorization of country was applied for a European‐wide study, TMAO levels in HF patients differed by region and a different association with outcome was observed for mortality risk.^31^

Notably, TMAO is just the ‘tip of the iceberg’ of one of the complex pathways involved in the association between the gut and the cardiac metabolism (i.e. short‐chain fatty acids, bile acids, and TMA‐TMAO).^1^ In this context, novel evidence showed that also other metabolites of the TMAO pathway (*Figure* *1*) are involved in HF with a promising prognostic role. Specifically, the carnitine‐related metabolites have shown associations with adverse outcomes in acute HF, in particular L‐carnitine and acetyl‐L‐carnitine for short‐term outcomes.^30^ In addition, data from the BIOSTAT‐CHF study showed that acetyl‐L‐carnitine, gamma‐butyrobetaine, and L‐carnitine were associated with outcome at 3 years, with a graded association when combined alone or with markers of gut dysfunction (i.e., a ‘panel of gut dysfunction’) with clinical status, NPs levels, and outcome.^21^

The importance of the association between GI and HF goes further with the identification of novel biomarkers for risk stratification.^3^ Considering that TMAO levels were not affected by recommended HF therapy, the ‘gut–HF axis’ may represent a novel field of interest for future research; specifically, a targeted therapy aimed to modify the gut microbiota could be a novel therapeutic approach. However, to date, no large‐scale trials have been performed to test this hypothesis.

In conclusion, data available from literature so far have shown that TMAO levels are increased in HF patients compared with healthy subjects and that elevated levels are associated with a poor prognosis. Although additional studies needed to address the current limitations in the field, TMAO, its precursors, and the gut‐derived biomarkers in general, represent a promising biomarkers of risk stratification with a possible role as a therapeutic target in the future.

## Conflict of interest

None.
